# Transcript profiling by microarray and marker analysis of the short cotton (*Gossypium hirsutum* L.) fiber mutant Ligon lintless-1 (*Li*_*1*_)

**DOI:** 10.1186/1471-2164-14-403

**Published:** 2013-06-17

**Authors:** Matthew K Gilbert, Rickie B Turley, Hee Jin Kim, Ping Li, Gregory Thyssen, Yuhong Tang, Christopher D Delhom, Marina Naoumkina, David D Fang

**Affiliations:** 1 Cotton Fiber Bioscience Research Unit, USDA-ARS, Southern Regional Research Center, New Orleans, LA 70124, USA; 2 Crop Genetics Research Unit, USDA-ARS, Mid South Area, Stoneville, MS 38772, USA; 3 The Samuel Roberts Noble Foundation, Genomics Core Facility, Ardmore, OK 73401, USA; 4 Cotton Structure and Quality Research Unit, USDA-ARS, Southern Regional Research Center, New Orleans, LA 70124, USA

**Keywords:** Cotton, Ligon Lintless-1, Microarray, Cotton fiber elongation, Microsatellite markers

## Abstract

**Background:**

Cotton fiber length is very important to the quality of textiles. Understanding the genetics and physiology of cotton fiber elongation can provide valuable tools to the cotton industry by targeting genes or other molecules responsible for fiber elongation. Ligon Lintless-1 (*Li*_*1*_) is a monogenic mutant in Upland cotton (*Gossypium hirsutum*) which exhibits an early cessation of fiber elongation resulting in very short fibers (< 6 mm) at maturity. This presents an excellent model system for studying the underlying molecular and cellular processes involved with cotton fiber elongation. Previous reports have characterized *Li*_*1*_ at early cell wall elongation and during later secondary cell wall synthesis, however there has been very limited analysis of the transition period between these developmental time points.

**Results:**

Physical and morphological measurements of the *Li*_*1*_ mutant fibers were conducted, including measurement of the cellulose content during development. Affymetrix microarrays were used to analyze transcript profiles at the critical developmental time points of 3 days post anthesis (DPA), the late elongation stage of 12 DPA and the early secondary cell wall synthesis stage of 16 DPA. The results indicated severe disruption to key hormonal and other pathways related to fiber development, especially pertaining to the transition stage from elongation to secondary cell wall synthesis. Gene Ontology enrichment analysis identified several key pathways at the transition stage that exhibited altered regulation. Genes involved in ethylene biosynthesis and primary cell wall rearrangement were affected, and a primary cell wall-related cellulose synthase was transcriptionally repressed. Linkage mapping using a population of 2,553 F_2_ individuals identified SSR markers associated with the *Li*_*1*_ genetic locus on chromosome 22. Linkage mapping in combination with utilizing the diploid *G. raimondii* genome sequences permitted additional analysis of the region containing the *Li*_*1*_ gene.

**Conclusions:**

The early termination of fiber elongation in the *Li*_*1*_ mutant is likely controlled by an early upstream regulatory factor resulting in the altered regulation of hundreds of downstream genes. Several elongation-related genes that exhibited altered expression profiles in the *Li*_*1*_ mutant were identified. Molecular markers closely associated with the *Li*_*1*_ locus were developed. Results presented here will lay the foundation for further investigation of the genetic and molecular mechanisms of fiber elongation.

## Background

Cotton seed fibers are single-celled trichomes that initiate from the ovule epidermal cells on or about the day of anthesis (DOA) [1]. Approximately 25% of the ovule epidermal cells differentiate into fiber cells during the initiation stage of cotton fiber development and subsequently undergo a period of rapid elongation known as the elongation stage [2,3]. The rate of fiber elongation peaks at approximately 6 to 12 days post-anthesis (DPA) and nears cessation around 22 DPA [4]. During peak elongation fiber cells can increase in length at rates of 2 mm / day or more depending on environmental factors and genotypes [5-7]. Beginning at 12–16 DPA and overlapping with the elongation phase is the secondary cell wall (SCW) biosynthesis stage. During this stage cellulose is synthesized and deposited between the primary cell wall and the plasmalemma [8,9]. The period of overlap between the elongation stage and the initial stage of SCW biosynthesis is referred as the transition period. Elongation and SCW biosynthesis continue until the fibers reach full length [25–30 mm in Upland cotton (*Gossypium hirsutum* L.) cultivars] [10], after which the cotton bolls open and the fibers desiccate under exposure to the environment. The environmental and genetic factors that influence the timing of these processes have been shown to also influence the development of desirable fiber traits such as lint yield and fiber quality [7,11-13].

Several naturally occurred cotton mutations affecting a range of fiber phenotypes have been genetically and functionally characterized in cotton. Examples include the completely glabrous seeds (lintless and fiberless) observed in MD17 [14], the fuzzless/lintless (*fl*) mutant of XZ-142 [15,16], and lines with seeds containing only lint and no fuzz, such as the naked seed lines *N*_*1*_ and *n*_*2*_[17]. Mutant lines exhibiting very short seed fibers include the Ligon Lintless-1 and −2 lines (*Li*_*1*_ and *Li*_*2*_) [18,19]. Recently, Cai et al. [20] analyzed a man-made mutant Lix that showed similar phenotype to *Li*_*1*_. The understanding that initiation, elongation, and secondary cell wall synthesis are distinct developmental processes often leads to the utilization of the applicable mutant to study the specific process of interest. For example, *fl* mutant seeds lacking any fiber emergence have served as models for studying initiation processes where enrichment of the homeodomain–leucine zipper transcription factor (*GhHD1*) and *GhMyb25* were identified as important for initiation [21,22]. Likewise, *N*_*1*_, with its lack of fuzz fiber and sparsely-distributed lint fibers has been used to characterize fiber elongation processes [17].

In a near-isogenic state with the cotton line Texas Marker-1 (TM-1), both the *Li*_*1*_ and *Li*_*2*_ mutants have seed fibers that are extremely short (< 6 mm) compared to wild type (WT) fibers that are typically greater than 20 mm in length [19,23,24]. As a monogenic dominant trait, the short-fiber phenotypes of *Li*_*1*_ and *Li*_*2*_ are identical in either a homozygous dominant or heterozygous state. Unlike the *Li*_2_ mutant which appears healthy and morphologically identical to the homozygous recessive wild-type plants with the exception of shorter seed fibers, the *Li*_*1*_ mutant exhibits pleiotropy in the form of severely stunted and deformed plants in both the homozygous dominant and heterozygous state [23].

Since the seed fibers of *Li*_*1*_ and *Li*_*2*_ fibers are shortened lint and fuzz fibers, these cotton mutants represent excellent candidates to study the molecular mechanisms of fiber elongation. Previously, our laboratory conducted extensive analysis of the *Li*_*2*_ mutant using microarray technology, molecular mapping and metabolomic analysis [25,26]. We developed microsatellite markers associated with the *Li*_*2*_ genetic locus, and identified transcripts or genes and metabolites that were affected by the *Li*_*2*_ mutation. In order to gain more comprehensive knowledge about cotton fiber development, and especially fiber elongation, we included the *Li*_*1*_ mutant as a subject of our investigation.

The *Li*_*1*_ mutant has been used as a model to study both primary and secondary cell wall processes [27-30]. However, previous microarray experiments with the *Li*_*1*_ mutant conducted during either very early elongation or later SCW stage failed to identify significant numbers of differentially expressed transcripts. For example, the microarray experiments conducted by Bolten et al.[28] using 24 DPA fibers only identified ~100 differentially expressed transcripts, notable among them *SuSy*, *Expansins*, and *Myb* transcription factors. However, apparent phenotypic differences in the *Li*_*1*_ as early as 3 DPA [31] indicating that altered gene expression may exist at or before this stage. Noting this, a microarray experiment conducted by Liu et al. [27] analyzed the *Li*_*1*_ mutant at the initiation and elongation stages of 0, 3 and 6 DPA. Their findings concurred with several earlier studies on the relevance of auxin, gibberellins, brassinosteroid and ethylene-related pathways in fiber development. Elongation stage (6 DPA) fibers from *Li*_*1*_ demonstrated a significant alteration in transcript profiles, with 1,398 target sequences showing altered expression in the mutant. Despite this, a crucial gap remains in our understanding of how the *Li*_*1*_ mutation affects the transcript profile at the transition period (later elongation stages and early SCW stages). This paper is the first attempt to analyze gene expression patterns in the *Li*_*1*_ mutant using microarray technology at these critical developmental stages. Here we provide a more complete picture of the molecular events directly controlling fiber elongation. Further, it will better define the mutation in terms of its effects on primary cell wall elongation and early secondary cell wall synthesis.

Prior research has determined that the *Li*_*1*_ gene is located on chromosome 22 using both SSR [31] and RFLP markers [32]. Karaca et al. [31] identified the SSR marker MP4030 that was 12.83 cM away from the *Li*_*1*_ locus. Rong et al. [32] provided the highest resolution to date, determining *Li*_*1*_ was flanked by RFLP markers Gate4CA09 and Coau1J04 at 2.7 and 1.3 cM away, respectively, based on 151 F_2_ progeny derived from an interspecific cross of *G. barbadense* Pima S-7 × *Li*_*1*_ mutant.

In order to conduct a comprehensive study of the *Li*_*1*_ mutant, we first created near-isogenic lines (NIL) in DP5690 genetic background by implementing an extensive backcross scheme. The use of DP5690, a modern variety, exhibits stronger growth characteristics than TM-1 in many climates and permits for additional analysis with the previously characterized Ligon Lintless-2 (*Li*_*2*_), which is also in the DP5690 background [25]. Using the two NILs as parents, we made a very large F_2_ population comprising 2,553 progeny which was used to identify molecular markers closely associated with the *Li*_*1*_ locus. To understand the molecular events that control fiber elongation and identify regulatory elements involved in this process, we obtained transcript profiles at 3 DPA (beginning of elongation), 12 DPA (late elongation stage) and 16 DPA (early SCW) using Affymetrix microarrays, and analyzed morphological characteristics of fibers from the *Li*_*1*_ NILs at different developmental stages. The objectives of this research were to determine the location of the *Li*_*1*_ locus on the chromosome, and identify genes that were differentially expressed during the development of WT and mutant *Li*_*1*_ fibers. This information will, in turn, be useful to identify the *Li*_*1*_ gene, and help to elucidate the molecular mechanisms of this gene on fiber elongation.

## Methods

### Plant materials for microarray and quantitative PCR (qPCR) experiments

**Figure 1:**
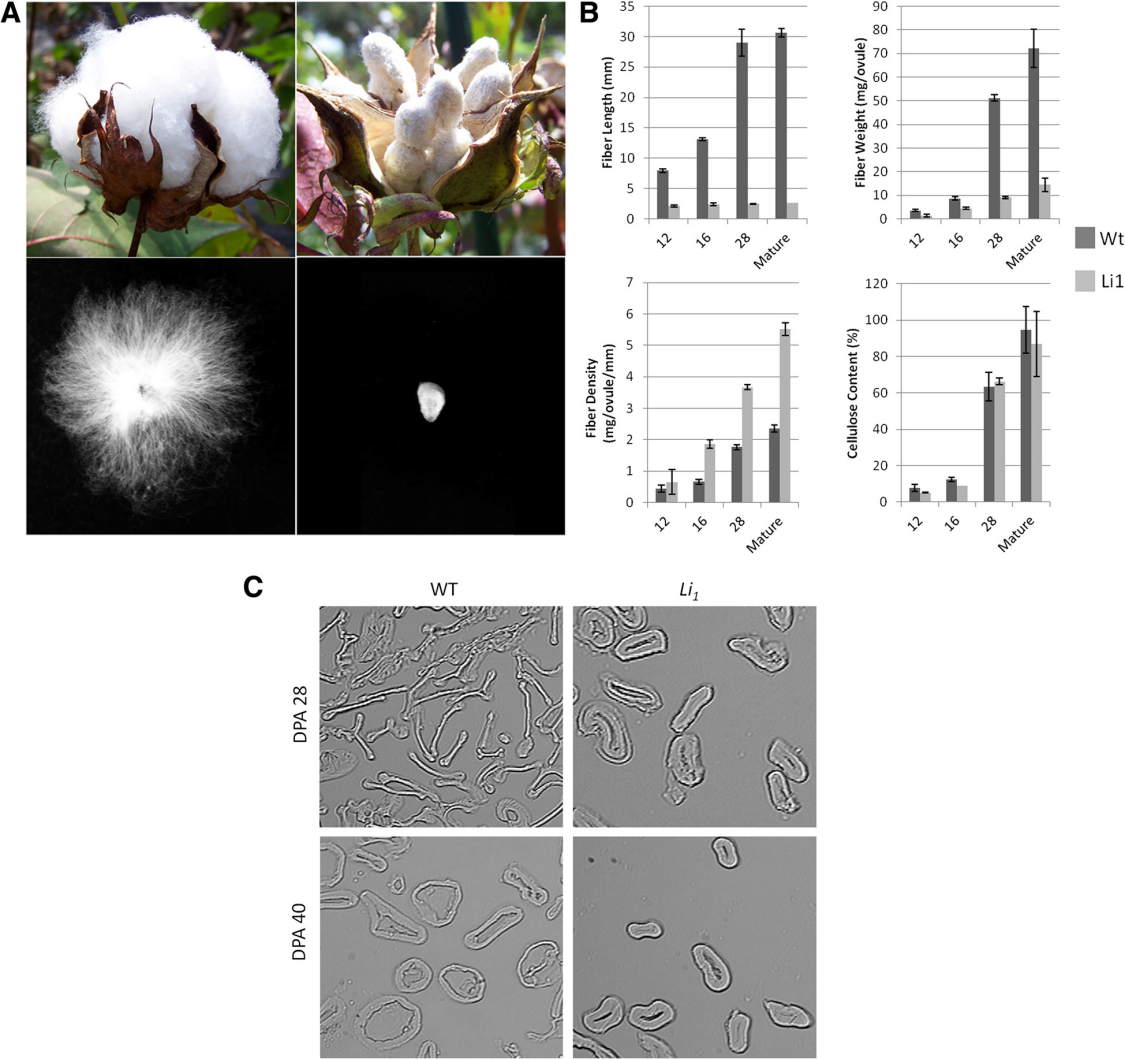
**Phenotype of the *Li***_***1 ***_**mutant and its wild type. A**) Comparison of phenotypes observed in wild type DP5690 and *Li*_*1*_/ *Li*_*1*_ mutant under field conditions after opening (top row) and of single individual seeds (bottom row). **B**) Measurements of fiber length, mass, density and fiber cellulose content in the wild type (DP5690) and *Li*_*1*_/ *Li*_*1*_ mutant. **C**) Cross-sectional images of fibers from 28 and 40 DPA wild type (WT) and *Li*_*1*_ mutant fibers (200× magnification).

Two near-isogenic lines of *Li*_*1*_ Upland cottons (*Gossypium hirsutum* L.) that were homozygous dominant (*Li*_*1*_*Li*_*1*_) and homozygous recessive (*li*_*1*_*li*_*1*_*)* for the *Li*_*1*_ locus were developed in a backcross program at Stoneville, MS in field and greenhouse environments (Figure 1A). Texas marker-1 (TM-1) cotton plants containing the *Li*_*1*_ gene were crossed with the Upland cotton variety DP5690. F_1_ progeny were backcrossed for five generations (BC_5_) by single seed decent (SSD) to DP5690 which served as the recurrent parent in each backcross. At the end of BC_5_ cycle, plants with *Li*_*1*_ phenotype were self-pollinated. The DP5690 recurrent parent was a pure inbred line that was self-pollinated for nine generations via SSD (Additional file 1). Progenies in each backcross were selected based on the phenotype for the *Li*_*1*_ short-fiber mutation.

For the microarray and qPCR experiments, a total of 100 homozygous (*Li*_*1*_*Li*_*1*_) *Li*_*1*_ mutant and 100 WT (*li*_*1*_*li*_*1*_) plants were planted in a field at the Southern Regional Research Center, New Orleans, LA in the summers of 2011 and 2012. The soil type in New Orleans was Aquent dredged over alluvium in an elevated location to provide adequate drainage. Flowers were tagged and sample collections were made before 10:00 am and immediately placed on ice. All samples collected within each developmental stage were tagged and collected on the same day. Fruits were randomly grouped into 3 individual replicates with 5–10 fruits per replicate. Fruits were then dissected, frozen in liquid nitrogen and stored at −80°C until further processing.

### Mapping population

A WT DP5690 (*li*_*1*_*li*_*1*_) was used as the female in a cross with its near-isogenic mutant line (*Li*_*1*_*Li*_*1*_ homozygous plant). Two thousand five hundred fifty-three F_2_ plants derived from approximately 20 F_1_ plants were planted along with their parents in a field in Stoneville, MS in 2012. The *Li*_*1*_ trait of each F_2_ progeny plant was evaluated after boll maturation and opening (about 60 DPA). Standard conventional field practices were applied during the growing season. The soil type in Stoneville, MS was Bosket very fine sandy loam.

### Fiber length and cellulose content measurements

Fiber length was measured using the method described by Schubert et al. [6]. Two replicate samples with 10 ovules each were measured. For mass determination, air-dried fibers were gently removed from all ovules of each sample, and weighted on an analytical balance. Cellulose content from each fiber sample was measured using the method described by Updegraff [33] with minor modification. Dried fiber samples were cut into small pieces. Ten mg of the blended fibers were placed in 5 mL Reacti-Vials™ (Thermo Fisher Scientific, Waltham, MA). Non-cellulosic materials in fibers were hydrolyzed with acetic-nitric reagent. The remaining cellulose was hydrolyzed with sulfuric acid and measured by a colorimetric assay with anthrone using Avicel PH-101 (FMC, Rockland, ME) as a cellulose standard. The average cellulose content for each fiber sample was obtained from two biological and three technical replications.

### Imaging analysis of fiber cross-sections

Fibers from 28 and 40 DPA were manually separated from the seed. After bundling the fibers together, a new razor blade was used to cut 2.5 – 3 mm of fiber from the end to be sectioned. This ensured a majority of fibers were cross-sectioned near the middle of the fiber. The fiber samples were embedded, thin-section cut, and photographed using the method previously described [34]. The images were taken using a Nikon Cambridge Quantimet 900 microscope at 200× magnification with a Hitachi KP-DSO camera.

### RNA isolation from cotton fibers

RNA was isolated as previously described [25]. Briefly, material was obtained from developing ovules using a glass bead shearing technique [35]. To separate the fibers from the ovules the samples were shaken vigorously enough to break fibers without damaging the ovules. Isolation of RNA was conducted using the Sigma Spectrum™ Plant Total RNA Kit (Sigma-Aldrich, St. Louis, MO) with on-column DNaseI digestion according to the manufacturer’s instructions. RNA quantity was determined by using a Nanodrop 2000 spectrophotometer (NanoDrop Technologies Inc., Wilmington, DE). A RNA integrity number (RIN) was determined for each sample using an Agilent Bioanalyzer 2100 and the RNA 6000 Nano Kit Chip (Agilent Technologies Inc., Santa Clara, CA). Only samples with RIN values of 7.0 or higher were used for further analysis.

### Microarray hybridizations and data analysis

The minimum information about microarray experiments (MIAME) guidelines were followed for all microarray experiments conducted in this study [36]. The microarray chips used for this study were the commercially available Affymetrix GeneChip® Cotton Genome Microarray (Affymetrix Inc., Santa Clara, CA), comprising 239,777 probe sets representing 21,854 cotton transcripts from a variety of EST databases. Labeling of the RNA was conducted using the Affymetrix GeneChip® 3′ IVT Express Kit and hybridizations were conducted according to the manufacturer’s protocols. Hybridizations were conducted on 3, 12, and 16 DPA samples with two biological replicates from each developmental stage. Data normalization and the determination of statistically relevant deviations in expression patterns were performed as described [37]. To assist in analysis of biological processes represented in the data, Gene-Ontology Enrichment Analysis (GOEA) was performed using the agriGO Singular Enrichment Analysis tool [38]. The statistical test method used was the Fisher’s Exact test (significance level 0.05). Annotation of the probe sets was accomplished with Blast2Go [39], and analysis of the cellulose synthase probes was conducted by translating all 6 reading frames of the probe sets and subjecting them to blastp analysis.

To investigate the activity of known cell elongation and cell wall-related genes, the microarray probe sets were compared with published lists of genes [27,40]. Each probe set reference sequence was aligned to the *G. raimondii* reference genome [41] with blastn and the best hit with e value of 1×10^-30^ or smaller was used to establish an annotation. The *G. raimondii* gene annotations specify an arabidopsis homolog for each gene, which was used to classify the functions of each Affymetrix probe set. Seventy-four probe sets were classified as elongation genes based on an earlier report [27]. To classify probe sets as primary or secondary cell wall genes, co-expression with arabidopsis microarray data with known primary or secondary cell wall cellulose synthase genes [40] was obtained from ATTEDII [42]. This strategy produced lists of 81 primary cell wall and 43 secondary cell wall gene probe sets.

### Reverse transcription and quantitative PCR

The cDNA reactions were performed using the iScript™ cDNA Synthesis kit (Bio-Rad Laboratories, Hercules, CA) per the manufacturer’s instructions. The reaction without reverse transcriptase served as negative control for testing genomic DNA contamination of the RNA samples. This reaction was then used as template in a qPCR reaction to verify that no amplification occurred. After cDNA synthesis, the qPCR reaction was conducted using iTaq™ SYBR® Green Supermix (Bio-Rad Laboratories) in a Bio-Rad CFX96 real time PCR detection system. PCR conditions and the protocols for determining primer efficiencies were as previously described [25]. Ubiquitin-conjugated protein (UCP) (Genebank AI730710) was used as the endogenous reference gene. Primer sequences are listed in Additional file 2.

### SSR marker analysis and genetic mapping

Young leaves were collected from each individual F_2_ plant and parents, and stored at −80°C. Total DNA was extracted from frozen leaves according to Fang et al. [43]. The *Li*_*1*_ gene was previously determined to reside on chromosome 22 (Chr. 22) [31,32]. To rapidly identify molecular markers closely linked to the *Li*_*1*_ locus, all simple sequence repeat (SSR) markers that were mapped on both Chr.22 and its homeologous Chr. 4 based on the high density consensus genetic map [44] were selected for analysis. RFLP markers reported by Rong et al. [32] were not screened due to unavailability of probes, and technical difficulties for RFLP marker analysis. The SSR markers mapped on Chr. 5 and its homeologous Chr.19 were also included because of a known translocation between Chr. 4 and Chr. 5 [45,46]. All together, a total of 921 SSR markers mapped in these four chromosomes were screened for polymorphism between DNA bulks. For the WT bulk, DNA of 10 F_2_ plants with WT phenotype were pooled at equal ratio and diluted to 50ng/μL. The mutant type bulk consisted of DNA from 10 F_2_ plants with short seed fiber. Four DNA bulks were made, two for each type. The polymorphic markers were then analyzed using 96 F_2_ progeny to identify markers closely associated with the *Li*_*1*_ locus. Only SSR markers that revealed less than 10 recombination events were analyzed among the total 2,553 F_2_ progeny plants.The PCR amplification conditions and marker data acquisition were according to Fang et al. [43]. All SSR primer sequences can be obtained from Cotton Marker database (http://www.cottonmarker.org). Segregation data for the *Li*_*1*_ trait and SSR markers were mapped using program JoinMap4.0 [47] with logarithm of odds score =25.

### Functional analysis of the *Li*_*1*_ region

To obtain sequences in the region determined to contain the *Li*_*1*_ mutant gene, the identified SSR marker sequences were aligned to the diploid *G. raimondii* D_5_ genome [41]. We also blasted other SSR marker sequences from the *Li*_*1*_ interval of Chr. 22 based on the high density consensus map [44]. This permitted comparison of the gene annotations for this interval with the annotations provided by Affymetrix to identify candidate genes. qPCR on select genes was conducted as described above.

### Sequencing introns to develop additional polymorphic markers

To identify additional polymorphisms that could facilitate finer mapping of the *Li*_*1*_ locus, we sequenced introns from the annotated genes in the 17 Mb interval between the markers TMB2500 and DPL0489 according to the reference *G. raimondii* genome sequences. Our rational to sequence introns was that introns tend to have higher sequence variations than exons. We designed primers that flanked 23 introns but were anchored in protein coding sequences. Three genes that resided in this interval and showed differential expression between WT and mutant were included. Amplicons were generated from total genomic DNAs of the parental lines and two F_2_ individuals (one WT type and one mutant type) and were Sanger sequenced.

## Results

### Fiber structure analysis

Early literature indicated that the *Li*_*1*_ mutant in a TM-1 genetic background demonstrated a reduced rate of crystalline cellulose deposition during primary cell wall synthesis and an increased rate during SCW synthesis, resulting in a “thickened” appearance of the cell wall in the mutant [18,30]. However, it remains unclear if this effect is due primarily to the mutation causing inhibition of fiber elongation processes or due to the mutation affecting SCW synthesis processes such as cellulose deposition, or both. To better characterize phenotypic changes in the mutant during late elongation and secondary cell wall deposition, changes in fiber length, mass, density, and cellulose content over developmental times were measured. Grown in standard field conditions in New Orleans, LA, the homozygous *Li*_*1*_ mutant demonstrated a characteristic short fiber phenotype and other previously characterized pleiotropic characteristics (Figure 1A and Additional file 3). Heterozygous individuals derived from DP5690 x *Li*_*1*_*Li*_*1*_ crosses demonstrated a dosage effect resulting in an intermediate plant size (Additional file 3). Measurements of fiber physical properties indicated that *Li*_*1*_ mutants had a dramatic difference in both length and fiber dry weight (mg/seed) at the developmental stages measured (Figure 1B) as compared to the WT. Elongation ceased or remained static in the *Li*_*1*_ mutant by 12 DPA, however fiber dry weight continued to increase during SCW synthesis and through to maturity, likely due to the continued deposition of cellulose. Calculating fiber density further illustrated the continued increase in biomass observed over the developmental stages and a much higher density in comparison to the WT. However, the overall cellulose content per unit mass was similar between WT and the *Li*_*1*_ mutant in 12, 16, 28 DPA and mature fibers. Image analysis indicated the *Li*_*1*_ mutant fibers at 28 DPA, (the youngest stage that was technically obtainable), were in general “thicker” than WT fibers (Figure 1C), which corroborated our observations on the increasing mass of the fibers. Combined, these results suggested that while thickening of the secondary cell wall occurred through developmental stages, it was not due to a relative increase in overall cellulose content or rate of production per fiber cell in the *Li*_*1*_ mutant. Rather, due to early cessation of fiber elongation, the stunted fiber did not distribute the cellulose along its longitudinal axis, thus resulting in a thickened appearance, and an increased mass per unit length.

### Microarray analysis

**Figure 2:**
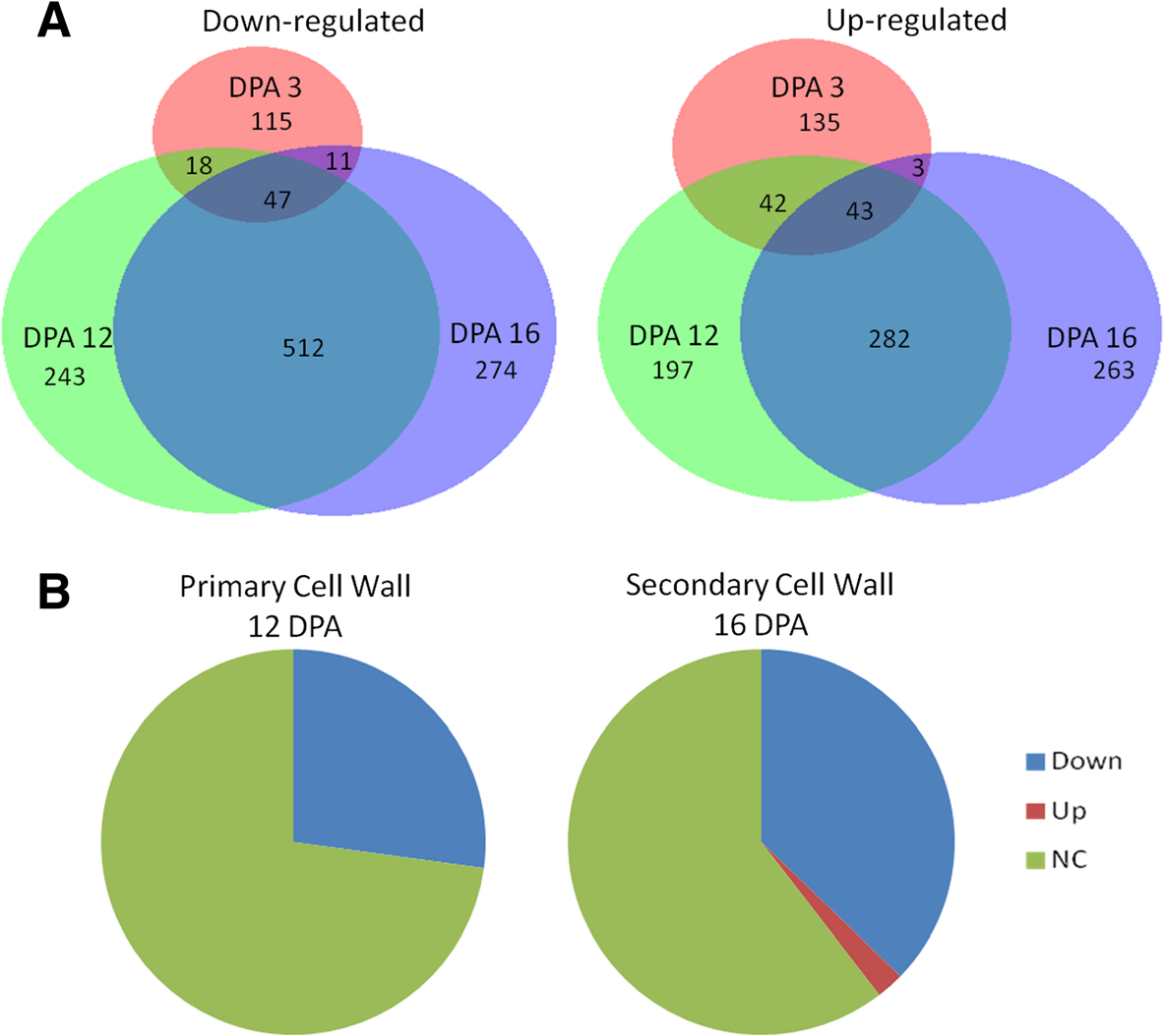
**Results of the microarray analysis comparing *Li***_***1 ***_**and its WT NIL in DP5690 background. A**) The distribution and number of probe sets from the microarray showing altered regulated by >2 fold in *Li*_*1*_. **B**) Pie diagram illustrating the relative percentages of probe sets showing altered regulation in the *Li*_*1*_ mutant that were identified as primary cell wall related in 12 DPA fiber samples and secondary cell wall related in 16 DPA fibers.

All three developmental time points analyzed demonstrated alterations in gene expression in the *Li*_*1*_ mutant. At 3 DPA the effects of the mutation were relatively limited, showing only a total of 223 genes up-regulated and 191 genes down-regulated (≥2-fold change; < Bonferroni-corrected p-value threshold 2.07194E-06). Of importance, 250 of these probe sets were unique to 3 DPA and were not differentially expressed at 12 or 16 DPA (Figure 2A). At 12 DPA and 16 DPA, 1,384 and 1,435 genes were differentially expressed, respectively. To analyze which developmental processes were affected in the *Li*_*1*_ mutant, target sequences previously identified as elongation, primary cell wall synthesis, or secondary cell wall synthesis were tabulated in each of the experimental categories. Figure 2B illustrates that more than a quarter of primary cell wall genes were down-regulated in the *Li*_*1*_ mutant at 12 DPA, as were a third of secondary cell wall biosynthesis genes at 16 DPA. Very few cell wall-related probe sets were up or down-regulated at 3 DPA (Additional file 4). This analysis revealed that while the *Li*_*1*_ mutation affected transcriptional activity at all stages of development, a major effect was the inhibition of primary cell wall-related factors in addition to a limited effect on secondary cell wall-related processes.

Gene Ontology Enrichment Analysis (GOEA) [38] of differentially expressed genes at 3 DPA indicated that no significant enrichment pertaining to a biological or molecular processes occurred at this stage in the *Li*_*1*_. GOEA analysis indicated that a large number of genes involved in known biological processes were detectable at 12 DPA, and many of these categories are known to be elongation-related processes, eg, xyloglucan endotransglucosylase [48], beta-galactosidase [49], shaggy-related kinase (*bin2*) [50], and auxin response genes [51] (Table 1). Importantly, there was essentially no enrichment of probes specific for any biological or molecular processes that showed altered regulation at 16 DPA only.

**Table 1:** **Gene ontology analysis results of target sequences that are up or down regulated in the *Li***_***1 ***_**mutant at 12 DPA or 16 DPA**

**Category**	Gene ontology categorization*	**Representative annotations of probed sequences (Li1/Wt expression ratio)**
Unique to 12 DPA	Response to hormone stimulus GO:0009725 (0.00023)	
		eg. indole-3-acetic acid-amido synthetase (0.48), xyloglucan endotransglucosylase (2.2), shaggy-related kinase (2.1), auxin-responsive protein (.34) and auxin response (0.42).
	Response to cytokinin stimulus GO:0009735 (0.00014)	
		eg. ap2 erf domain-containing transcription factor (2.1), homeobox protein knotted-1-like 3-like (2.1)
	Transmembrane receptor protein tyr kinase signaling pathway GO:0007169 (1.9e-06)	
		e.g. strubbelig-receptor family 7 protein (0.45), lrr receptor-like serine threonine-protein kinase (0.35),
	Protein amino acid phosphorylation GO:0006468 (1.5e-05)	
		e.g. cyclin-dependent kinase f-4-like (0.48), shaggy-related protein kinase eta-like (2.07), serine threonine-protein kinase aurora-1 (.43), feronia receptor-like kinase (0.43)
	Hydrolase activity, hydrolyzing O-glycosyl compounds GO:0004553 (3e-07)	
		e.g. cobra-like 4 protein (3.74), beta galactosidase 1 (0.46), xyloglucan endotransglucosylase hydrolase (0.44), acidic chitinase (3.32), xyloglucan endotransglucosylase hydrolase protein 2 (2.33), beta-xylosidase alpha-l-arabinofuranosidase 2-like (0.43)
	Xyloglucan:xyloglucosyl transferase activity GO:0016762 (0.00016)	
		e.g. xyloglucan endotransglucosylase hydrolase (0.41), xyloglucan endotransglucosylase hydrolase protein 2 (2.33), probable xyloglucan endotransglucosylase hydrolase protein 32-like (2.56)
	Protein kinase activity GO:0004672 (0.00075)	
		serine threonine-protein kinase (3.12), probable receptor-like serine threonine-protein kinase at5g57670-like (11.02)
Unique to 16 DPA	Cellular nitrogen compound metabolic process GO:0034641 (1.1e-05)	
		glutamine synthetase (2.05), phenylalanine ammonia-lyase (0.26), asparagine synthetase (2.06), serine threonine protein kinase 2 (3.23)

*e-value in parenthesis.

A significant number of processes that were common to 12 DPA and 16 DPA, but not to 3 DPA showed altered expression in the mutant (Additional file 5). GOEA analysis of this category of genes indicated a large decrease in probe sets categorized in nucleosome assembly (GO:0006334) and lipid transport (GO:0006869). There was an increase in mitochondrial electron transport (GO:0006120), which includes NADH-dehydrogenase genes and NADH-plastoquinon oxidoreductase subunits, both members of Complex I of the electron transport chain, known to be high producers of reactive oxygen species (ROS) [52].

Fiber development-related genes show altered expression patterns in the *Li*_*1*_ fibers (Table 2, more comprehensive list in Additional file 6). A large number of auxin, ethylene, and gibberellins responsive transcription factors were differentially expressed, as were cytoskeleton components such as tubulin and profilin. Five probe sets identifying 1-Aminocyclopropane-1-Carboxylic Acid Oxidase genes, responsible for ethylene biosynthesis, were differentially expressed. Ghi.798.1, identified as *GhACO3*, demonstrated significant up-regulation in the mutant, as did an uncharacterized ACO homologue (GhiAffx.16665). However, *GhACO4* (Ghi.8025) and another uncharacterized *ACO* homologue (Ghi.6502) were down-regulated in the mutant, suggesting divergent roles in fiber elongation processes. Another key enzyme in ethylene biosynthesis, 1-Aminocyclopropane-1-carboxylate synthase (*ACC*) [53,54], was down-regulated in the mutant at 12 DPA. Ghi.5451 shares 100% homology with ACC Synthase 3 and 6 (*GhACS 3/6*) (Table 2).

**Table 2:** **Select elongation-related probe sets showing altered regulation in the *Li***_***1 ***_mutant*

**Genes up-regulated in the*Li***_***1***_**mutant relative to its near isogenic wild type line**						
**Probe ID**	**Annotation**	**3DPA**	**12DPA**	**16DPA**	**Heterologous functions**	**Ref.**
Ghi.798.1.S1_s_at	1-aminocyclopropane-1-carboxylate oxidase	**2.45**	**10.95**	**5.08**	*GhACO3*, ethylene biosynthesis, elongation related	[55]
GhiAffx.16665.1.S1_s_at	1-aminocyclopropane-1-carboxylate oxidase homolog 4-like	1.48	**2.87**	**2.54**	no homology known	
Ghi.8264.1.S1_s_at	brassinosteroid-regulated protein bru1	0.81	**4.11**	**6.99**	homology to xyloglucan endotransglcosylase (AT4G14130.1), expression correlates with elongation inhibition	[56]
Ghi.5860.1.S1_s_at	fasciclin-like arabinogalactan protein	1.10	**56.47**	**0.16**	EST from fiber, homology to *G. hirs.* fasciclin-like arabinogalactan protein 3 (FLA3). *AtFLA3* overexpression leads to defective elongation in stamen.	[57]
GhiAffx.36662.1.S1_s_at	r2r3-myb transcription	1.60	**4.25**	**6.40**	At4g37260, MYB73. Highly responsive to ethylene, ABA. Downregulated in *fl* mutant elonagtion phase.	[58,59]
GhiAffx.60562.1.S1_at	ethylene-responsive transcription factor wri1	0.79	**0.31**	**0.26**	*wri1-1* mutants demonstrate defective elongation of hypocotyl.	[60]
Ghi.2039.2.S1_s_at	sucrose synthase sus1	**35.38**	1.60	1.38	*GhSus1* isoform C, targeted to cell wall during secondary cell wall synthesis.	[61]
Ghi.7911.1.S1_x_at	xyloglucan endotransglucosylase hydrolase	**0.42**	**9.24**	**8.22**	TCH4, dwarf *A. thaliana* mutants have reduced expression, elongation related.	[62]
GhiAffx.10621.1.A1_s_at	pollen ole e 1 allergen and extensin family protein	1.21	**32.53**	0.67	Uncharacterized EST	
**Genes down-regulated in the*Li***_***1***_**mutant relative to its near isogenic wild type line**						
Ghi.8025.1.S1_s_at	1-aminocyclopropane-1-carboxylate oxidase	1.37	**0.36**	**0.32**	*ACO4*, ethylene biosynthesis, induced in ovule culture by fatty acid synthesis, increasing ethylene production and root elongation.	[55,63]
Ghi.6502.1.S1_at	1-aminocyclopropane-1-carboxylate oxidase	1.13	0.63	**0.14**	EST, significant homology to ACO proteins, but not identical to ACO1-4	
Ghi.5451.1.S1_at	1-aminocyclopropane-1-carboxylate synthase	0.78	**0.47**	0.88	Highest homology with *GhACS 3/6*	
GhiAffx.12577.1.S1_at	gibberellin 20-oxidase	0.95	**0.34**	**0.15**	gibberellin synthesis, stimulated by Auxin treatment in *A. thaliana,* which also causes hypocotyl cell elongation. Increased expression in transgenic cotton causes increased fiber length.	[64-66]
Ghi.8087.1.S1_s_at	myb-like transcription factor 3	1.32	**0.18**	**0.16**	GhMYB3, contains gibberellin responsive GLABROUS1, which promotes trichome formation in A. thaliana.	[67,68]
Ghi.10822.1.S1_at	xyloglucan endotransglucosylase hydrolase	1.10	**0.41**	0.52	*GhXTH2*, high homology to coding region of *GhXTH1* which produces longer fibers when over-expressed in transgenic cotton.	[69]
GraAffx.28354.1.S1_s_at	rho gtpase activation protein	0.52	**0.48**	0.78	High homology to ROP1, tip-localized GTPase responsible for cell elongation and polarity.	[70]

*Bold and underlined indicates significant at the Bonferroni-corrected 0.05 probability level for microarray data.

Twelve probe sets representing an unknown number of AP2/ERF (ethylene response factor) domain-containing transcription factors were significantly differentially expressed, with the majority demonstrating down-regulation (e.g. Ghi.7874) (Table 2 and Additional file 6). Eight probe sets measuring levels of the pathogenesis-related 10 (PR10) proteins, a family of defense and stress-related genes regulated by jasmonic acid, ethylene and other effectors [71] exhibited expression patterns 8 to 96 fold higher in the *Li*_*1*_ mutant. *Sucrose Synthase 1* (*Sus1*) was highly up-regulated (56-fold) at 3 DPA in the *Li*_*1*_ mutant, then not differentially expressed at 12 DPA and 16 DPA.

### Transcription analysis of cellulose synthase activity

**Figure 3:**
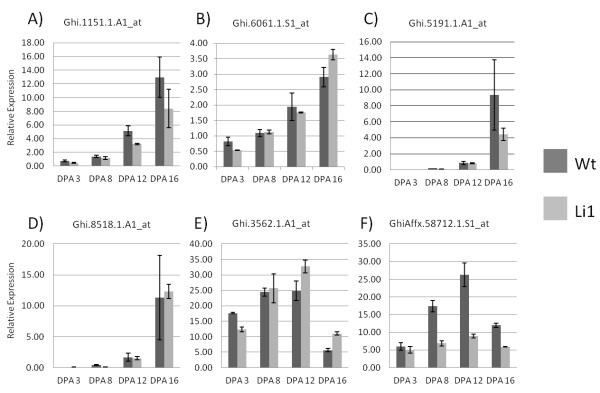
**Cellulose synthase expression profiles in wild type and *Li***_***1 ***_**mutant as determined by qPCR analysis. A**) Ghi.1151 (GhCesA1), **B**) Ghi.6061 (GhCesA2), **C**) Ghi.5191 (GhCesA3) and **D**) Ghi.8518 (GhCesA5) demonstrated expression patterns consistent with secondary cell wall synthesis and did not exhibit altered regulation in the *Li*_*1*_ mutant. **E**) Ghi.3562.1 (Cellulose synthase-like E1, CSLE1) also did not demonstrate altered gene expression in the *Li*_*1*_ mutant, however showed expression consistent with being primary cell wall-related. **F**) GhiAffx.58712.1 is identified as GhCesA6 (ACS88358), showed significant decreased expression in the *Li*_*1*_ mutant at the mid to late elongation stages (8 and 12 DPA).

Cellulose synthases and cell wall re-arranging proteins have been a subject of interest as they relate to fiber elongation. More specifically, defining the role of specific cellulose syntheses in so far as their specificity for either primary or secondary cell wall processes is relevant to understanding elongation. Previous research has speculated, and some data has suggested, that altered cellulose deposition determined the *Li*_*1*_ phenotype [18,30]. In light of this, it was of value to further analyze cellulose synthase expression levels in the *Li*_*1*_ mutant. Probe sets annotated as cellulose synthase (*Ces*) genes or cellulose synthase-like (*Csl*) were identified by Blast2Go annotation, Affymetrix-provided annotation terms, and by blastx analysis of the microarray probe sets. Quantitative PCR analysis was conducted on selected *Ces* genes. Of the 38 probe sets annotated as *Ces* or *Csl*, only 1 probe set, GhiAffx.58712 demonstrated reproducible >2 fold altered gene expression in the *Li*_*1*_ mutant at the developmental stages analyzed (Figure 3). Probe set GhiAffx.58712 sequence was derived from one of 28 EST’s homologous to sequence accession [GenBank:GQ200733]. In the WT the target sequence expression levels were relatively high at 8 DPA and 12 DPA, then decreased at 16 DPA indicating potential function as a primary cell wall-related *Ces* (Figure 3F). Importantly, the *Li*_*1*_ mutant showed decreased expression levels relative to WT at the elongation stages analyzed. GhiAffx.58712.1 was identified as GhCesA6 [GenBank:ACS88358], which shares 86% protein sequence identity with *A. thaliana* CesA6 [GenBank:NP_201279], which is known to be important in primary cell wall synthesis [72]. Other sequences analyzed; Ghi.1151 (GhCesA1), Ghi.6061 (GhCesA2), Ghi.5191 (GhCesA3), Ghi.8518 (GhCesA5) and Ghi.3562.1 (Cellulose synthase-like E1, CSLE1) did not demonstrate altered expression in the *Li*_*1*_ mutant (Table 2 and Figure 3A-E).

### Corroboration of microarray data

Sequences targeted by the microarray were selected for quantitative PCR analysis to corroborate the results obtained in the microarray (Table 3). The selected sequences included genes that were up-regulated, down-regulated, and demonstrated no change in the *Li*_*1*_ mutant. The 24 samples analyzed (8 probe sets, 3 developmental stages) by qPCR demonstrated results consistent with the microarray analysis. The probe sets targeting cellulose synthase-like protein e6-like demonstrated little alteration in gene expression in the *Li*_*1*_ mutant according to the microarray data, and similar results were obtained with qPCR analysis. One of the eight probe sets targeting pathogenesis-related protein 10 family (Ghi.6485) exhibited a dramatic increase in expression, showing 127 fold and 191 fold increase in 12 DPA and 16 DPA, respectively. Expansin a10, a cell wall structural protein, was down-regulated at 12 and 16 DPA in microarray and qPCR, as were most expansins (Additional file 6). Expansin-like b1 was the only expansin up-regulated at 12 DPA, however the qPCR data for this gene was not entirely consistent with microarray data for 16 DPA. Beta-galactosidase, which hydrolyses β-glycosidic bonds and is thought to be important for primary cell wall rearrangement, was down-regulated as measured by qPCR and microarray in all stages of development in the mutant. TubulinA4 was expressed at extremely high levels (data not shown) although differential regulation between WT and the *Li*_*1*_ mutant was not significant.

**Table 3:** **Microarray expression ratios of *Li***_***1***_/WT and corroboration by RT-qPCR analysis*

		**3DPA**		**12DPA**		**16DPA**	
		**Micro-array**	**RT-qPCR**	**Micro-array**	**RT-qPCR**	**Micro-array**	**RT-qPCR**
GhiAffx.58712.1.S1_at	cellulose synthase catalytic subunit	0.93	**0.84**	**0.39**	0.34	**0.51**	**0.49**
Ghi.3562.1.A1_at	cellulose synthase-like protein e6-like	**0.49**	0.70	**1.17**	1.32	**2.24**	1.94
Ghi.5057.1.S1_s_at	protein wax2	**0.47**	0.41	**0.59**	0.46	**0.42**	0.29
Ghi.6485.1.S1_s_at	pathogenesis-related protein 10	**1.34**	1.51	**70.42**	**127.01**	**77.04**	**191.91**
Gra.3004.2.S1_s_at	expansin a10	0.82	0.82	**0.46**	**0.38**	**0.46**	0.33
Ghi.6465.2.S1_at	expansin-like b1	0.99	0.40	**6.32**	**6.74**	**10.12**	0.37
Gra.2056.1.A1_s_at	beta-galactosidase 13	**0.23**	**0.12**	**0.09**	**0.05**	**0.17**	0.11
Ghi.1314.1.S1_x_at	Tubulin alpha 4 (Tua 4)	**0.91**	0.57	**0.88**	0.49	1.02	0.80

*Bold and underlined indicates significant at the Bonferroni-corrected 0.05 probability level for microarray data and at the 0.05 probability as determined by a two-tailed t-test for qPCR data.

### Mapping the *Li*_*1*_ locus region with SSR markers

**Figure 4:**
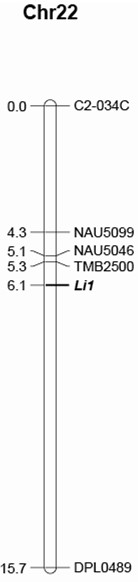
**Linkage map of *Li***_***1 ***_**genetic locus region on Chr. 22.** The distances (cM) are indicated on the left of the map and marker names on the right.

Of the 2,553 F2 progeny, 1,604 showed the Li_1_ mutant phenotype, and 949 were WT. This segregation deviated significantly from a single dominant-gene model (*χ*^*2*^ = 605) presumably due to the failure of many homozygous mutants (which had deformed stems and leaves with stunted plants) to germinate or survive as suggested by Rong et al. [32] and Liu et al. [73]. Of the 921 SSR markers screened, 12 (1.3%) were polymorphic between two DNA bulks. Of them, 7, 3, 1, and 1 were previously mapped on Chr.22, Chr.19, Chr.4 and Chr.5, respectively based on the high density consensus map [44]. Analysis of these 12 markers among 96 F2 progeny indentified only 5 markers that were associated with the *Li*_*1*_ locus, and mapped on Chr.22. These 5 markers were further evaluated in the whole 2553 F_2_ progeny plants. A map was constructed around the *Li*_*1*_ region (Figure 4). The marker TMB2500 was 0.8 cM away from the *Li*_*1*_ locus.

A total of 13 kb sequences from the introns of 23 genes residing in the 17 Mb interval between markers TMB2500 and DPL0489 were compared between the WT and *Li*_*1*_ mutant. However, we were unable to identify additional sequence polymorphisms to facilitate finer genetic mapping of the *Li*_*1*_ locus.

### Functional analysis of the *Li*_*1*_ region

**Figure 5:**
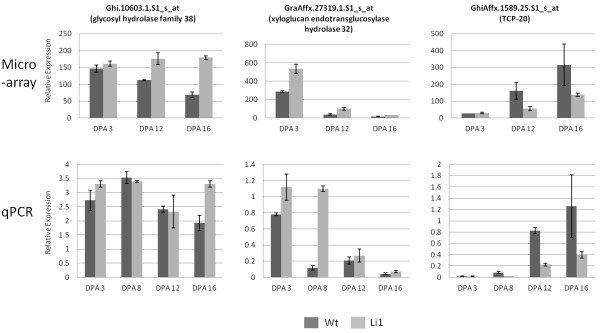
**Transcript profiles of select genes near the *Li***_***1 ***_**locus.** Microarray (top row) and qPCR analysis (bottom row) of select genes found in the interval of Chr. 22 containing the *Li*_*1*_ locus. Probes with high homology to a glycosyl hydrolase (Ghi.10603.1.S1_s_at), xyloglucan endotransglucosylase/hydrolase 32 (GraAffx.27319.1.S1_s_at) and TCP20 (GhiAffx.1589.25.S1_s_at) were selected due to the altered regulation in the *Li*_*1*_ mutant, proximity to the mutation, and potential role in elongation.

Based on the mapping results, genes within the 17-Mb region between the best hits of the flanking SSR markers TMB2500 and DPL0489 in the *G. raimondii* reference genome were further analyzed. Eighty probe sets from the microarray corresponded to genes that were in this interval, of which 24 showed altered expression in the *Li*_*1*_ mutant (Additional file 7). Three of these genes were further evaluated by qPCR analysis to establish a link between the mapping and expression data. Ghi.10603.1.S1_s_at, GraAffx.27319.1.S1_s_at, GhiAffx.1589.25.S1_s_at have homology with glycosyl hydrolase family protein 38 (E = 5e-103), xyloglucan endotransglucosylase/hydrolase 32 (E = 5e-150), and AtTCP20 (E = 1e-42), respectively. The expression profile of the glycosyl hydrolase 38 showed altered expression in the mutant at 16 DPA by both microarray and qPCR (Figure 5). Xyloglucan endotransglucosylase was up-regulated at early and mid elongation stages (3 DPA and 8 DPA), and according to the microarray data remained slightly elevated at 12 DPA. The transcription factor TCP-20 was up-regulated at the late elongation stage of 12 DPA and 16 DPA.

## Discussion

A previous microarray experiment examining 0 and 3 DPA fibers from the *Li*_*1*_ mutant found little evidence of global or significant alterations in gene expression patterns [27]. Conversely, microarray data of 6 DPA fibers and proteomic analysis of 12 DPA from *Li*_*1*_ fibers demonstrated significant perturbation of expression profiles in the mutant, indicating that processes related to primary cell wall elongation are affected in the *Li*_*1*_ mutant [27,29]. In addition, earlier studies of the *Li*_*1*_ mutant focusing on secondary cell wall synthesis failed to report findings of upstream processes being significantly affected [28]. Thus this analysis on late elongation and early SCW stages would complement and extend these earlier findings aimed to better characterize the effects of the mutation at both primary and secondary cell wall synthesis, and provide data for the fiber development during the transition period. The morphological and molecular data presented here supports the model that the *Li*_*1*_ mutation is an upstream factor primarily targeting elongation processes. Several ontological categories of genes and individual genes that have previously been identified as having a role in fiber elongation were identified, as were new targets for investigation.

Previous proteomic analysis of *Li*_*1*_ at 12 DPA [29] identified a limited number of proteins that could be corroborated by our microarray data. A comprehensive analysis revealed that both data sets include the down regulation of cytoskelatal tubulin proteins and metabolism-related proteins (ie. glycolipid transfer protein and a pyrophosphatase). The stress response proteins nucleoredoxin and germin-like proteins, a flavanone-3-hydroxylase and translation factor 6 were increased in both data sets. However, the remainder of proteins identified as showing altered regulation were not corroborated by our data. A detail list is shown in the Additional file 8. This could be due to the difference in genetic background, or due to the technical limitations of 2-dimensional gel electrophoresis. Analysis of more recent Affymetrix microarray data obtained at 6 DPA [27] is limited by the fact that the authors did not provide probe set information, thus preventing a more rigorous or comprehensive analysis from being conducted. However, they also identified several actin and tubulin genes that were down-regulated in the mutant. At 6 DPA there was an increase in *xyloglucan endotransglucosylase*, fascilin-like arabinogalactan protein, and the ethylene synthesis-related 1-aminocyclopropane −1-carboxylate oxidase (*GhACO3*) which our data indicated remained true at 12 DPA. As expected several differences also exist. Analysis of 6 DPA fibers showed decrease expression levels of auxin-IAA-related genes and the ethylene responsive AP2/ERF family genes, which then became up-regulated in the mutant at 12 DPA. Conversely, another gene involved in ethylene production, *GhACO4,* was up-regulated at 6 DPA but down-regulated in the mutant at 12 DPA. Genes that were down-regulated in *Li*_*1*_ at 6 DPA, but were at WT levels in 12 DPA were enriched in plasma membrane associated proteins (GO:0005886, p = 0.0008), such as filament-like plant protein 4, myosin heavy chain, and perk1-like protein kinase.

The use of the *Li*_*1*_ mutant as a model system for both elongation and secondary cell wall synthesis was based on earlier studies utilizing *Li*_*1*_ in a TM-1 genetic background that measured the ratio of fiber weight to length and showed an increase in fiber mass throughout development, implicating continued or increased cellulose deposition [18]. Despite its very short fibers, the authors claimed the dry mass of the *Li*_*1*_ fiber was approximately 83% of its NIL. A second study using the same genetic line that measured [^14^C] glucose deposition in the secondary cell wall indicated a 5-fold higher rate of cellulose deposition per mm of fiber in the *Li*_*1*_ mutant [30]. However, much of our data using *Li*_*1*_ in a DP5690 genetic background suggested that molecular events involved in cellulose deposition and secondary cell wall synthesis were not affected to the degree that primary cell wall processes were. Measurements indicated that the dry mass of the fiber was approximately 20% of its NIL and that the actual cellulose content per unit mass remained unchanged (Figure 1B). Further, qPCR of cellulose synthase genes showed that secondary cell wall genes remained largely unaffected. These results support the model that thickening of the cell wall is due to inhibited elongation processes, but not due to increased cellulose production. The “increased rate” of glucose incorporation observed by Kohel et al. [30] was based on measurements of cellulose production per unit length instead of per unit mass. However, since elongation ceases in the *Li*_*1*_ mutant but its cellulose production continues at WT levels, we speculate that *Li*_*1*_ fibers expand outwardly, thus resulting in an increased cellulose production per unit length. It is worthy to mention that the use of a different NIL in our study as compared with previous studies may also account for differences in fiber measurements.

The ontological categories of the 434 unique probes differentially expressed at 12 DPA included Xyloglucan:xyloglucosyl transferase activity (GO:0016762) (Table 1). Xyloglucan endotransglycosylase/hydrolase (XTH) enzymes are proposed to disrupt xyloglucan-cellulose crosslinks, thus permitting cell wall rearrangement and fiber elongation [48]. Six probe sets identified as XTH showed differential regulation in the *Li*_*1*_ mutant. One probe set (Ghi.7911.1) was down-regulated at 3 DPA and significantly up-regulated (9 fold) at 12 DPA and 16 DPA (Additional file 6). This gene [GenBank:AY476737] remains uncharacterized in *Gossypium*, however its homeologous gene in *A. thaliana* TCH4/XTH22 (TAIR;AT5G57560) demonstrated very similar results in a study conducted on petiole elongation that indicated XTH22 is under different genetic regulation than other characterized XTH’s [74]. Of the remaining XTHs identified in our microarray data, three probe sets exhibited decreased expression at 12 DPA and one slightly increased. While it is clearly plausible that a decreased expression of XTHs could lead to the inhibition of elongation, the role of XTHs that increased in the *Li*_*1*_ mutant warrants further investigation. Other ontological categories affected in the mutant included “responses to hormone synthesis” (GO:0009725) (ie. IAA synthetase and auxin-response genes) and “hydrolase activity, hydrolyzing O-glycosyl compounds” (GO:0004553), which contain 19 probe sets likely correlated with hormonal activation to signal entry into the primary to secondary cell wall transition stage [75]. The role of IAA, auxin response genes, and hormonal regulatory enzymes such as glucosyltranferases in fiber development processes has been widely documented [75]. It is of interest to note here that the only category identifying this set of related genes was the list of probes that were up or down-regulated only at 12 DPA. Probe sets that were also enriched or decreased in 16 DPA mutant tissues (Additional file 5) were not enriched in these ontological categories, implying that altered hormonal regulation in the mutant was occurring primarily before and at 12 DPA. Analysis of hormone contents by Chen et al. [76] identified altered levels of Abscisic Acid, dihydrozeatin, and others in the *Li*_*1*_ mutant, but only analyzed up to 8 DPA. Microarray data on 6 DPA fibers reported altered expression of several hormone related peptides in the *Li*_*1*_ mutant, such as auxin-related genes, gibberellins, brassinosteroid, abscisic acid and jasmonic acid-related genes [27]. These data suggest, when taken together with molecular and morphological data, that the early cessation of elongation in *Li*_*1*_, is in part due to the culmination of altered hormonal factors that occurs during early to mid elongation.

Consistent with what has been reported at earlier developmental stages, multiple genes related to ethylene biosynthesis and ethylene response were differentially expressed in the *Li*_*1*_ fibers [27]. A key enzyme in ethylene biosynthesis, 1-aminocyclopropane-1-carboxylate synthase (ACS), hybridized by probe set Ghi.5451and demonstrated a 2-fold down-regulation at 12 DPA (Table 2). Previous studies have correlated increased ACS activity with cotton fiber elongation [54]. 1-aminocyclopropane-1-carboxylate oxidases (ACO) 1–4 are also involved in ethylene biosynthesis [54]. *ACO3* (Ghi.798.1) showed a significant up-regulation at all stages analyzed, and *ACO4* (Ghi.8025.1) exhibited down-regulation at 12 DPA and 16 DPA. *ACO1* and *ACO2* (Ghi.6953.1 and Gra.2141.1, respectively) did not show altered expression in the *Li*_*1*_ mutant. Consistent with our data, previous studies have implicated *ACO3* as having peak expression at the late elongation stage near 12 DPA. *ACO1* and *2* have demonstrated different expression patterns with peak expression in early secondary cell wall synthesis stage [54,55], implying that they may be involved in later developmental stages. The fact that *ACO3* was related to elongation in previous studies and showed altered regulation in our microarray (in contrast to *ACO1* and *2*) further supports *Li*_*1*_ being a key elongation-related mutation.

Pathogenesis-related protein 10 family of proteins (PR10) exhibited a significant alteration in their expression patterns in the *Li*_*1*_ mutant, ranging from 8 to 96 fold difference between *Li*_*1*_ and WT. This family of proteins consists of a large and functionally diverse group of proteins, ranging in function from antimicrobial/antiviral activity, hormone/ligand binding, secondary metabolism, and abiotic stress response (reviewed in [77]). Further implying a role in growth and development, individual PR10 proteins have been found to be regulated by multiple phytohormone-related cis-regulatory sequences including ethylene response elements [78] and brassinosteroids [79]. However it remains unclear if the stress response seen in the *Li*_*1*_ is related to previously mentioned ROS levels or a response to altered hormone expression.

The cellulose synthase activity reported here is of particular interest. Previous reports have speculated that an increase in secondary cell wall cellulose synthase activity may account for the thickened cell wall of the *Li*_*1*_[30], however, our data failed to confirm this. Rather, another *GhCes*, probed by Ghi.58712, demonstrated altered expression at the elongation stage of development and showed an expression pattern consistent with elongation-related activity in the WT. Translation of the probe target’s consensus sequence and TAIR blast search showed the Ghi.58712 target sequence shared highest homology, although not identity with *AtCesA9,-2,-5, -6* and −*3* (E = 0.0). *AtcesA9* is only expressed during embryogenesis, however the remaining *AtCesA2, -5, -6*, and −*3* are members of a primary cell wall associated cellulose synthase complex (CSC) [72,80]. Substantial evidence exists that these closely related genes are cell elongation related. *Atces2* null mutations showed a severe dwarf phenotype in *A. thaliana*, and functional studies have demonstrated that *AtCesA2* and −*6* were partially functionally redundant during elongation [80]. *A. thaliana* mutants at the PROCUST1 locus, which encodes *cesA6*, exhibited cell elongation effects in a pleiotropic manner [81]. *cesA6* promoter-GUS fusion experiments have demonstrated that its expression occurs throughout the hypocotyl and root, peaking in the cell elongation zone of the expanding root [72].

The *Li*_*1*_ genetic locus was previously identified as residing on chromosome 22 [31,32]. Our results confirmed this chromosomal assignment. Based on the high density consensus genetic map constructed by Blenda et al. [44], it could be seen that the *Li*_*1*_ locus might be close to the centromeric region as original indicated by Rong et al. [32]. It is worthy of mention that the genetic distances observed in our experiment were larger than those reported by Rong et al. [32]. This greater recombination observed in our research might be due to our much larger (151 *vs* 2,553) population size and different population structure (interspecific *vs* intraspecific cross). There is a large gap (9.6 cM) between the *Li*_*1*_ locus and marker DPL0489 (Figure 4). We have screened all the SSR markers mapped in this interval based on the high density consensus map [44] and a newly published map [82], and could not further close the gap. Additionally, we were unable to identify sequence polymorphisms among the 23 gene introns that were located in the region harboring the *Li*_*1*_ locus even though some of these genes demonstrated differential expression between WT and mutant. This also indicates that altered gene expression may not be necessary due to gene sequence change. This result may also imply that the genomic region harboring *Li*_*1*_ locus is highly monomorphic. Recently, Cai et al. [20] reported a similar phenomenon when mapping the Lix locus. One of their flanking markers, NAU3469, was 24.5 cM away from the Lix locus.

The mapping data coupled with the recently released *G. raimondii* sequences provided the opportunity for additional analysis of sequences in the vicinity of the *Li*_*1*_ locus. Probe sets from the microarray with high homology to a glycosyl hydrolase family 38, a xyloglucan endotrans-glucosylase hydrolase 32, and AtTCP-20 are near the *Li*_*1*_ locus, demonstrated differential expression in the *Li*_*1*_ mutant and have apparent associations with elongation processes. In addition to the already discussed XTH enzymes, glycosyl hydrolase family 38, a family of related mannosidases, affect cell wall phenotypes when mutated in *A. thaliana*[83]. Interference with AtTCP-20 *in planta* in *A. thaliana* by fusion with a repressor domain resulted in severe developmental phenotypes characterized by reduced cellular elongation [84]. Additionally, a transcription factor identified in *Gossypium barbadense* with a highly homologous TCP domain, GbTCP, produced a short fiber phenotype when silenced by RNAi [85].

Determining which, if any of these is the *Li*_*1*_ mutation is currently under investigation. Next-generation sequencing of transcripts by RNA-seq is currently under way in our laboratory, and may reveal SNPs or splice variants responsible for either altered regulation of an elongation-specific gene, or a mutation resulting in a nonfunctional protein. Identification of these variations will facilitate developing closer markers, and help to eventual cloning of the *Li*_*1*_ gene.

The identification of the gene responsible for the *Li*_*1*_ phenotype would provide an invaluable tool in the quest to understand fiber elongation processes. In the meantime, the data generated here, in combination with published data from other developmental time points, has the potential to provide a sound basis for the examination of key hormonal, structural, and other pathways involved in cotton fiber elongation.

## Conclusions

Measurements of fiber characteristics and microarray analysis of the *Li*_*1*_ mutant and its WT were conducted at 3, 12, and 16 DPA with the goal of enhancing our understanding of cotton fiber elongation. Both methodologies supported the notion that the early cessation of elongation in *Li*_*1*_ was due to disruption of primary cell wall elongation-related processes. Further, we identified and discussed several elongation-related genes that exhibited altered expression profiles in the *Li*_*1*_ mutant, including a putative primary cell-wall related cellulose synthase. We conducted SSR marker analysis on a large population, and using the *G. raimondii* reference sequence identified elongation-related genes near the *Li*_*1*_ locus with altered expression levels. The data here will contribute to developing a comprehensive understanding of cotton fiber elongation.

### Availability of supporting data

The data sets supporting the results of this article are included within the article and its Additional files.

## Abbreviations

CSC: Cellulose synthase complex; DOA: Day of anthesis; DPA: Days post-anthesis; EST: Expressed sequence tag; GOEA: Gene ontology enrichment analysis; IVT: In-vitro transcription; NIL: Near-isogenic line; PCW: Primary cell wall; RFLP: Restriction fragment length polymorphism; RT-qPCR: Reverse transcription quantitative polymerase chain reaction; SCW: Secondary cell wall; SNP: Single nucleotide polymorphism; SSR: Simple sequence repeat; TM-1: Texas Marker 1; WT: Wild type.

## Competing interests

The authors declare that they have no competing interests.

## Authors’ contributions

DDF conceived the experiment, coordinated and supervised the research, identified molecular markers and conducted linkage mapping. MKG had the main responsibility for the study including the field work, tagging and sample harvest; RNA isolations and assessment of RNA quality; gene selection for corroboration of the microarray results; RT-qPCR and statistical analysis of the RT-qPCR data; and analyzing the microarray results. RBT developed the *Li*_*1*_ mutant and WT NILs and the F_2_ mapping population used as plant materials. HJK conducted fiber measurements and cellulose synthase assays. PL assisted with the molecular marker analysis. GT and MN assisted in sequence analysis. YT performed statistical analysis on the microarray data. CDD conducted fiber imaging analysis. MKG and DDF wrote the manuscript. All authors read and approved the final manuscript.

## Supplementary Material

Additional file 1 **Pedigree of the Li1 mutant and WT NILs.***Li*_*1*_*Li*_*1*_ and *li*_*1*_*li*_*1*_ were created using a *G. hirsutum* pure inbred cv. DP5690 backcrossed for 5 generations to a F_1_ generation DP5690*/Li*_*1*_. Click here for file

Additional file 2 **qPCR Primer Sequences.** Primer sequences used for quantitative PCR analysis. Click here for file

Additional file 3 ***Li***_***1***_***Li***_***1***_***, Li***_***1***_***li***_***1***_***and li***_***1***_***li***_***1***_**(WT) plants.** Image of wild type (DP5690) (left), a heterozygous *Li*_*1*_*/li*_*1*_ plant (center) and homozygous (*Li*_*1*_*/Li*_*1*_) (right) grown in standard field conditions and harvested five months after planting. Click here for file

Additional file 4 **Distribution of cell wall-related genes based on microarray data.** The relative distribution of elongation, primary cell wall and secondary cell wall related probe sets and there relative expression in the *Li*_*1*_ mutant in the developmental stages analyzed. Click here for file

Additional file 5 **Gene Ontology Enrichment Analysis for probe sets unique to 12 and 16 DPA.** Gene Ontology Enrichment Analysis for the probe sets that show altered regulation in both 12 DPA and 16 DPA fibers but exclude 3 DPA. Click here for file

Additional file 6 **Putative fiber-related probe sets and microarray data.** List of putative fiber-related genes that show altered expression in the *Li*_*1*_ mutant at 3, 12, and 16 DPA. Click here for file

Additional file 7 **Microarray results for targeted genes near the*Li***_***1***_** locus.** List of probe sets showing altered expression in the *Li*_*1*_ mutant that were determined to be near the *Li*_*1*_ locus. Click here for file

Additional file 8 **Transcript comparison between the present research and prior studies.** List of probe sets that were identified in prior studies and their status in the present research. Click here for file
