# Supplementary figures and images for: Transcript profiling by microarray and marker analysis of the short cotton (Gossypium hirsutum L.) fiber mutant Ligon lintless-1 (Li1)
Source: BMC Genomics. 2013 Jun 17;14:403. doi: 10.1186/1471-2164-14-403 (PMC3701525; doi:10.1186/1471-2164-14-403)

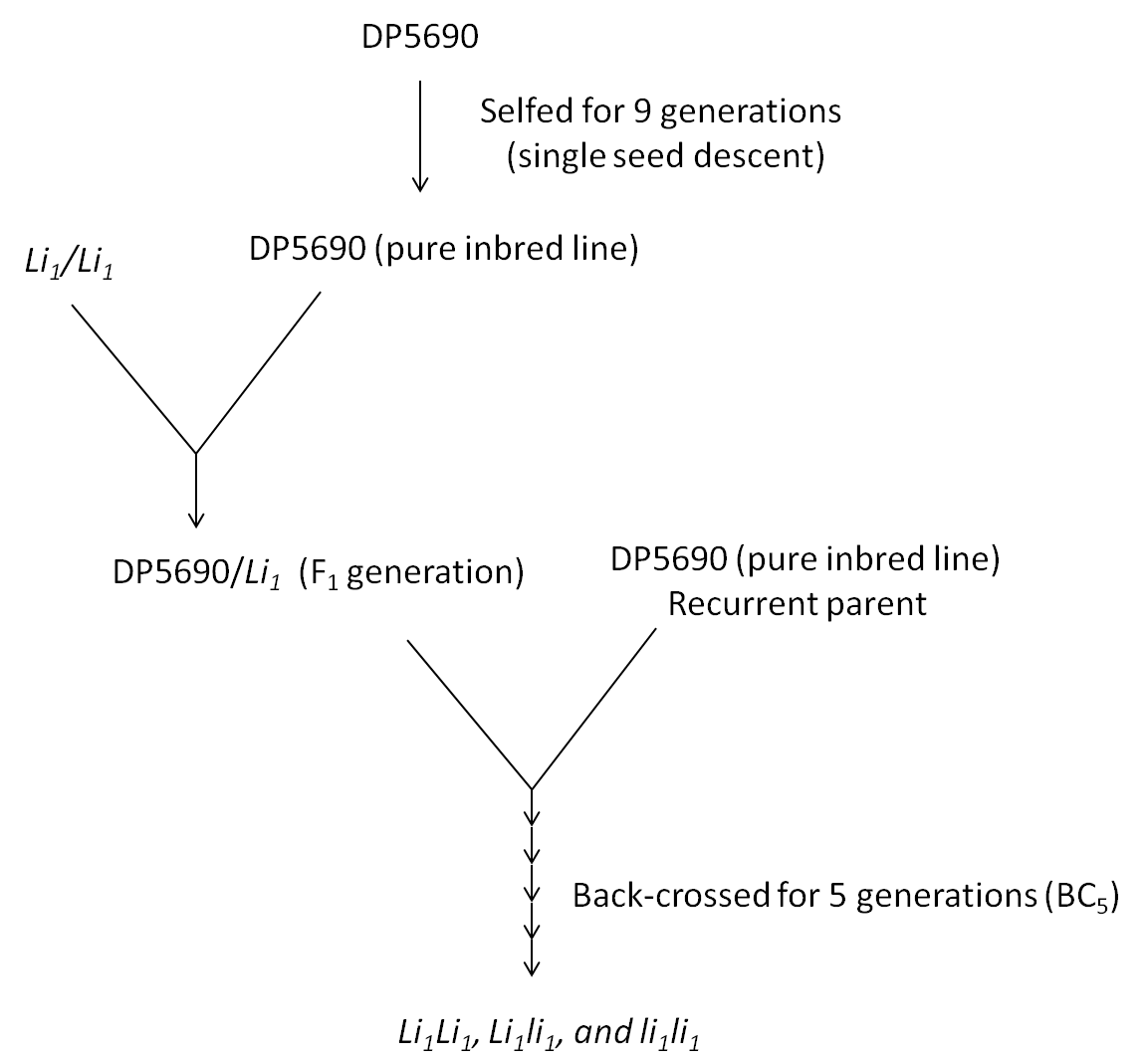

Supplement: Additional file 1 — Pedigree of the Li1 mutant and WT NILs.Li1Li1 and li1li1 were created using a G. hirsutum pure inbred cv. DP5690 backcrossed for 5 generations to a F1 generation DP5690/Li1. [file 1471-2164-14-403-S1.tiff]

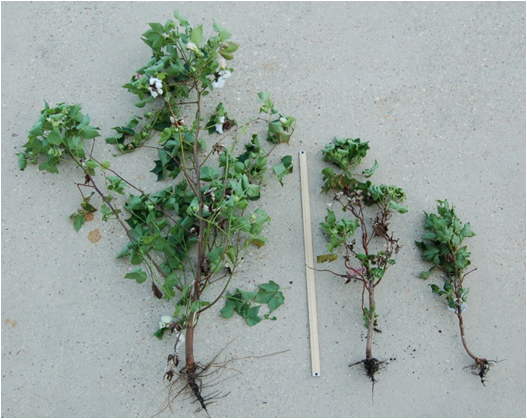

Supplement: Additional file 3 — Li1Li1, Li1li1and li1li1(WT) plants. Image of wild type (DP5690) (left), a heterozygous Li1/li1 plant (center) and homozygous (Li1/Li1) (right) grown in standard field conditions and harvested five months after planting. [file 1471-2164-14-403-S3.tiff]

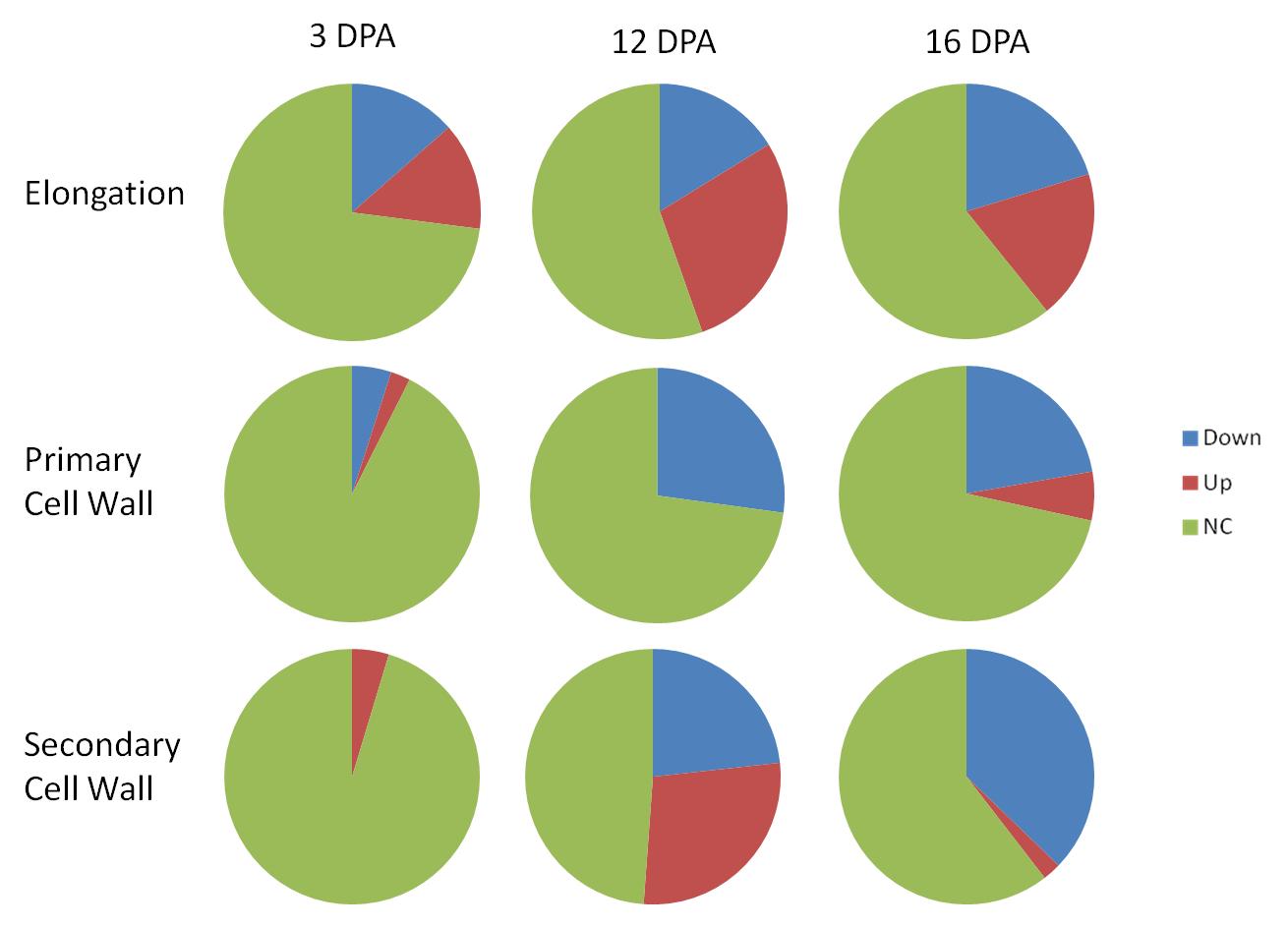

Supplement: Additional file 4 — Distribution of cell wall-related genes based on microarray data. The relative distribution of elongation, primary cell wall and secondary cell wall related probe sets and there relative expression in the Li1 mutant in the developmental stages analyzed. [file 1471-2164-14-403-S4.tiff]
